# The Acute Contagious Diseases of Childhood

**Published:** 1901-09

**Authors:** 


THE ACUTE CONTAGIOUS DISEASES OF CHILDHOOD. By MARCUS P. HATFIELD, A. M., M. D., Professor Emeritus of Diseases of Children, Northwestern University Medical School; Professor of Diseases of Children, Chicago Clinical School; Attending Physician, Wesley Hospital. Pages 142 Chicago: G. P. Engelhard & Co. 1901. [Price, $1.00 net.]

Dr. Hatfield presents a little book which contains chapters on scarlatina, measles, rötheln, mumps, whooping cough, varicella, variola, and influenza. Of each disease some account of the history, clinical history, prognosis and treatment, prophylaxis, pathology and bacteriology are given. The author in the preface says that no attempt to write an original work has been made, but rather a compilation from various writers, especially the later French and German pediatricians, has been attempted. The compiler's own experience is drawn upon, however, and with profit. None of the topics discussed are gone into deeply, but the volume is clearly written and very readable.

